# Driving to Contract Management in Health Care Institutes of Developing Countries

**Published:** 2012-04-01

**Authors:** S Vatankhah, O Barati, M R Maleki, Sh Tofighi, S Rafii

**Affiliations:** 1 Department of Health Services Management, Tehran University of Medical Sciences, Tehran, Iran; 2 National Institute of Health Research, Tehran University of Medical Sciences, Tehran, Iran; 3 Health Management Research Center, Baghiatallah University of Medical Sciences, Tehran, Iran

**Keywords:** Contract management, Developing country, Public, Private, Hospital, Government

## Abstract

**Background:**

Public hospitals can privatize management activities by contracting with a private organization or person to perform the work. Management contract is a method which uses private sector for major government projects like hospitals. This study evaluates contract management in health care institutes of developing countries.

**Methods:**

Information has been collected by reviewing the management contract condition of selected countries. Different forms of public private partnership for private participation in hospitals were surveyed.

**Results:**

The effects of management contract is expanding market opportunities to include public sector clients, capturing a market to be protected from competitors and providing a reliable and timely source of revenue.

**Conclusion:**

Contracting with non-governmental entities will provide better results than government provision of the same services. Contracting initiatives must be regulated and monitored at the highest level of government by experienced and astute policy makers, economists and operational personnel.

## Introduction

Since the early 1980s, public sector hospitals around the world have come under intense scrutiny in policy circles due to bureaucratic complexity of these institutions, the heavy burden they impose on public funds and the perceived difficulties in ensuring their efficient and effective functioning under centralized governmental control.[1] One policy option is delegation by contract as abroad privatization strategy. In this strategy, government can privatize an activity by contracting with a private organization to perform the work.[2]

As part of ongoing public services reform and in line with the objective of providing quality and affordable health care in an equitable manner, it is possible to expand the scope and improve the quality of health care delivery. These would include management outsourcing of hospital through private sector participation.[3]

One of the contractual forms in community non-profit hospitals is turning to management by other management firms as a means of achieving the benefits of multi-institutional arrangements. This method of administration greatly improves organizational performance.[4]

Management contract is a method which uses private sector for major government project like hospitals. The private sector designs and manages facilities for the lifetime of the contract and in this way; the risk of failing to deliver services to time and budget would transfer to the private sector. The management contracts are for design, construction and management of all or part of a hospital and require contracts to report on their performance through a formal performance system. Government uses this to charge deductions for failures to meet specified standards.[4][5]

Contract management is essential to ensure that services are delivered to specified standards and the price expected. Strong contract management is necessary to negotiate amendments to the contract in line with trusts' needs and to seek continuous improvement in performance and cost efficiency.[5][6]

The implementation of contract management can be described as a three step process. These steps overlap each other but they can be separately dealt with. The first step is to simply get the basic contract management operations established. These include such as a centralized contract repository, appointment of person (s) responsible for each contract, a contract management handbook, company contract templates and defining proactive alarms for the contracts.

The second step is to make the contracts alive in other words, be part of the business. This covers issues such as contract management process, deeper involvement business units, use of contracts in operation such as project management and active use of contract management as a business tool.

The third step in the process of contract management is the strategic step. As this level of development is achieved, an enterprise is able to evaluate contract and partnership network from a strategic point of view.[7][8] This study evaluates contract management in health care institutes of developing countries.

## Materials and Methods

In this study, information has been collected by reviewing the management contract condition of selected countries. The inclusion criteria for reviewing the above countries were: Having public hospitals, outsourcing medical services with the form of contact management and their success in implementing this method according to World Bank Report. The countries under study were: South America, India, Zimbabwe and Poland. For gathering data, the data bases of Tehran University of Medical Sciences, World Bank and World Health Organization were used. In this study, different forms of public private partnership for private participation in hospitals were surveyed. Also the effects and limitations of management contract in public and private sector perspective were studied (Table 1). For purposes of this investigation, system was defined as two or more hospitals that were owned, leased, sponsored or contract managed by a separate administrative entity. The investigation aims to survey different forms of public private partnership for private participation in hospitals, also to study the effects and limitations of management contract in public and private sector perspective.

**Table 1:** The effects and limitations of management contract.[10][11]

**Effects and limitations of management contract**	**Public sector perspective**	**private sector perspective**
Effects	By introducing competitive forces, contracting puts pressure on both public and private providers in terms of both service and price.	Contracting expands market opportunities to include public sector clients or customers.
	Expanding the range of activities subject to contracting encourages the private sector to expand the services it provides in areas desired by government.	Contracting allows a private provider to capture a market to be protected from competitors at least for the term of the contract.
	Contracting both requires and promotes better planning and policy development by improving the flow of information about volumes of goods, services, costs, quality, responsiveness, population served, health needs and other issues.	Contracting can provide a reliable and timely source of revenue, encouraging investments necessary to support quality and innovation and ensuring longer term viability.
	Contracting provides government with a mechanism for purchasing needed health services at an agreed on and therefore predictable price.	The contract term may provide adequate time to justify investments in the capital and personnel needed to support contractual obligations.
	Contracting promotes transparency, constrains conflicts of interest and reduces opportunity for graft and corruption.	
Limitations	Qualified private providers may not be available.	The costs of entering the market to compete successfully for government contracts may be too high.
	Qualified bidders may not be interested in contracting with government.	The market may be too unstable or the contract period too short to support needed capital investments.
	Competition may be weak or non existent.	Transaction costs, the costs of securing and managing the contract may be too high.
	Government may discourage bidders by shifting too much risk to providers.	There may be no legal resource if government fails to fulfill its contractual obligations.
	Successful contractors could compete with the public sector for health personnel.	
	Governmental capacity to negotiate contracts may be weak.	
	Government's managerial and bureaucratic overhead may increase.	
	Government may agree to disadvantages contract terms.	
	Long term contracts may restrict government flexibility.	
	Costs may be higher than anticipated.	

## Results

The Effects of management contract can be mentioned in seven categories as follows:

A. The national Audit Office declares that the performance of management contract typically meets the contractual specification. Sixty seven percent of trusts rated all services provided by management contract satisfactory. Of the 72 trusts that rated provided data, 15% rated all their services as better than satisfactory. The majority of trusts reported consistent or improved performance over the course of the contract.

B. After management contract, there was no change in traditional efficiency indicators like bed occupancy or average length of stay. However, many hospitals recorded significant improvements in management, finance and accounts, inventory control and general maintenance. Technical efficiency seems to be improved mainly due to the increased availability of supplies and improvements in building and equipment maintenance also the beneficial impact of these factors on staff productivity.

C. The impact on quality was limited to improvements in overall supply position. Supply and main-tenance of equipment also improved, increasing the availability of usable equipment.

D. Management contract has made the process of financial accountability more transparent in most countries but it has had little effect on public accountability for the nature and quality of services provided by the hospital.

E. Contract management hospitals had higher patient day costs but the average length of stay was shorter. Total cost per patient stay was slightly less than in traditionally managed hospitals. Private contractors can also avoid public service restrictions that interfere with efficient personnel management.

F. The average annual spend on estates maintenance was higher in management contract hospitals. These contracts require the building to be maintained to a high standard and to pay for maintenance over the life of the contract. The average cost of patient catering services was cheaper in management contract hospitals, although performance scores did not suggest a significant difference in the quality provided. The average cost of cleaning and laundry services delivered under management contract differed from the average costs of the same services at other hospitals.

G. But the differences were not statistically significant. Private contractors had greater management flexibility than state departments. They could respond faster to changing circumstances and tend to be more innovative.[9]

The findings suggest that with health care as well as other public services, communities across the country were struggling to build market oriented strategies into the delivery of public services without abandoning their commitment to serve those who may be left behind by the market.

All the studies found that contracting yielded positive results and showed that the contractors were more effective than the government, on the basis of several measures related to both quality of care and coverage of services.[7][8][9]

## Discussion

Contract management is a general concept that may encompass a wide range of operational, strategic and administrative components. This variation may include differences in management goals, array of services provided, specialty emphasis, depth of management experience and centralization of decision making in the management organization.

With the methodological concerns about the cases studied, there is still a need for more survey to include rigorous evaluations. However, the current weight of evidence suggests that contracting with non-governmental entities will provide better results than government provision of the same services. Far from limiting government involvement in health care, contracting may be one way of keeping publicly financed health care relevant. Governments in developing countries are currently responsible for only a modest role in providing curative services and contracted provision is a well established model for delivering health care services.[8][9][10]

Use of contracting is growing in many developing countries while the requirement of financial resources constrains its use, private sector interest in remuneration enables a substantial portion of the burden of interaction to be shifted out of government hands. The active cooperation of the contracted providers makes contracting less onerous to implement than regulation in many settings.[11]

Contracting in health care is diverse in terms of the types of actors that use it, the types of contractual relationships that are established and the purpose thereof. However, one must never lose sight of the fact that contracting is a tool that should be evaluated on the basis of its impact on the performance of a health system and on peoples' health. Contracting should not be reduced to a mere management tool used to cut health costs. It is an approach that should lead the various actors to offer to the public health services that are increasingly efficient, effective, superior and fair.

To ensure that contracting is used strategically to introduce market mechanisms while ensuring that essential public services are provided and the needs of poor and vulnerable are protected, the government needs to recognize that contracting is a powerful process, a purposeful methodology not just a cluster of independent transactions. This means that contracting initiatives must be regulated and monitored at the highest level of government by experienced and astute policy makers, economists and operational personnel. In fact, despite contracting limitations, there are some issues to be considered: The first is the provider payment mechanism, second, the nature of provider and the type of contract and finally, the impact of factors outside the contract itself.[12][13] Contracting with non-governmental entities will provide better results than government provision of the same services. Contracting initiatives must be regulated and monitored at the highest level of government by experienced and astute policy makers, economists and operational personnel.
